# Understanding Interventions to Address Infodemics Through Epidemiological, Socioecological, and Environmental Health Models: Framework Analysis

**DOI:** 10.2196/67119

**Published:** 2025-03-24

**Authors:** Jennifer N John, Sara Gorman, David Scales

**Affiliations:** 1 Perelman School of Medicine, Penn Medical Communication Research Institute, University of Pennsylvania, Smilow Center for Translational Research Room 12-136, 3400 Civic Center Boulevard, Philadelphia, PA, 19104, United States, 1 (215) 573-5359; 2 Critica, Bronx, NY, United States; 3 Department of Medicine, Division of General Internal Medicine, Weill Cornell Medicine, Cornell University, New York, NY, United States

**Keywords:** infodemics, misinformation, disinformation, Covid-19, infodemic management, health communication, pandemic preparedness

## Abstract

**Background:**

The COVID-19 pandemic was accompanied by a barrage of false, misleading, and manipulated information that inhibited effective pandemic response and led to thousands of preventable deaths. Recognition of the urgent public health threat posed by this infodemic led to the development of numerous infodemic management interventions by a wide range of actors. The need to respond rapidly and with limited information sometimes came at the expense of strategy and conceptual rigor. Given limited funding for public health communication and growing politicization of countermisinformation efforts, responses to future infodemics should be informed by a systematic and conceptually grounded evaluation of the successes and shortcomings of existing interventions to ensure credibility of the field and evidence-based action.

**Objectives:**

This study sought to identify gaps and opportunities in existing infodemic management interventions and to assess the use of public health frameworks to structure responses to infodemics.

**Methods:**

We expanded a previously developed dataset of infodemic management interventions, spanning guidelines, policies, and tools from governments, academic institutions, nonprofits, media companies, and other organizations, with 379 interventions included in total. We applied framework analysis to describe and interpret patterns within these interventions through their alignment with codes derived from 3 frameworks selected for their prominence in public health and infodemic-related scholarly discourse: the epidemiological model, the socioecological model, and the environmental health framework.

**Results:**

The epidemiological model revealed the need for rigorous, transparent risk assessments to triage misinformation. The socioecological model demonstrated an opportunity for greater coordination across levels of influence, with only 11% of interventions receiving multiple socioecological codes, and more robust partnerships with existing organizations. The environmental health framework showed that sustained approaches that comprehensively address all influences on the information environment are needed, representing only 19% of the dataset.

**Conclusions:**

Responses to future infodemics would benefit from cross-sector coordination, adoption of measurable and meaningful goals, and alignment with public health frameworks, which provide critical conceptual grounding for infodemic response approaches and ensure comprehensiveness of approach. Beyond individual interventions, a funded coordination mechanism can provide overarching strategic direction and promote collaboration.

## Introduction

### Background

The COVID-19 pandemic entailed an outbreak not only of viral illness but also of viral rumors. This so-called infodemic, defined by the World Health Organization as an overabundance of accurate and inaccurate information [1], had tangible public health consequences. As of April 2022, 24% of COVID-19 mortality, or 234,000 deaths, was vaccine-preventable [2], and misinformation and disinformation cost the United States between US $50,000,000 and US $300,000,000 each day during the pandemic in health care spending and economic losses [3]. These impacts demonstrated the necessity of addressing misinformation as part of public health responses [4].

A wide range of stakeholders globally including governments, nongovernmental organizations, academic institutions, professional societies, and technology companies rapidly developed and deployed a large number of interventions to mitigate the perceived harms of the infodemic. These interventions varied substantially in their foci and impacts and addressed both the infodemic itself and the social problems related to the infodemic, such as vaccine hesitancy and institutional distrust. For example, in the New York City Department of Health and Mental Hygiene, the misinformation response unit disseminated culturally specific communication materials in response to emerging web-based COVID-19 rumors through partnerships with community organizations [4]. YouTube and Google also prioritized credible health information sources in search results based on criteria developed by organizations including the World Health Organization, the National Academy of Medicine, and the Council of Medical Specialty Societies [5,6].

Given the inevitability and growing threat of future infodemics, it is critical to learn from the successes and shortcomings of the growing body of infodemic management interventions. Prior studies have evaluated the effectiveness of these interventions, their fundamental characteristics, and the psychological concepts underlying them [7-9]. However, these studies were limited in the scope of interventions examined, only considered 1 framework, or focused on individual-level factors. Little research has explored the areas of emphasis, both intended and unintended, and strategies revealed and gaps left by these interventions in aggregate. Such an analysis is needed to provide funders, government agencies, public health leaders, and other stakeholders that set priorities for infodemic responses with insights to inform proactive, sustainable, and coordinated efforts that effectively use limited resources. Given increasing politicized attacks on public health and misinformation research in recent years, it is particularly important to avoid infodemic management practices that lead to or exacerbate public mistrust. For example, in the United States, Republicans are disproportionately likely to consider the removal of false articles on social media, a key component of Facebook’s COVID-19 misinformation policy [10], to be censorship [11].

In public health, conceptual frameworks serve as lenses that systematically illuminate gaps, patterns, and opportunities in programs and policies [12-14]. Frameworks are not exhaustive or mutually exclusive, and multiple frameworks are necessary to comprehensively interrogate complex topics. Applying public health frameworks to infodemic interventions offers an opportunity to explore their theoretical foundations and inform the design of future interventions. Certain public health metaphors, particularly analogies to epidemics of disease, are frequently invoked in and often dominate discussions of misinformation in academia and public media. However, the use of these frameworks and the validity of their underlying assumptions in this setting have yet to be rigorously evaluated [15]. As a result, other promising mechanisms of impact supported by alternative paradigms may be overlooked [15]. In the following sections, we outline the 3 frameworks applied in this study and their applications to infodemics. These frameworks were selected because they are well established in public health or are often referenced, implicitly or explicitly, in infodemic-related discourse. Public health frameworks were prioritized to reflect the growing application of public health perspectives to address misinformation during the pandemic.

### Epidemiological Model

**Figure 1.**
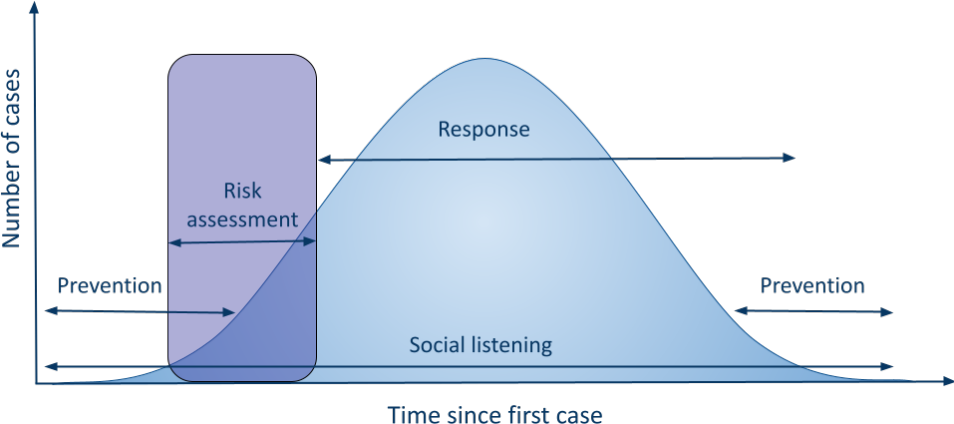
Epidemiological model.

Epidemiological models describe the spread of disease over time within a population. The epidemiological model frames misinformation as a contagion (Figure 1) [16]. As the epidemiological model is currently a dominant paradigm in discourse about misinformation [15], it is critical to assess how well suited previously developed interventions are to this model. Areas of engagement in the information ecosystem are drawn analogously from responses based on public health approaches to infectious diseases: social listening, risk assessment, response, and prevention [17]. Risk assessment can take place either as a one-time evaluation or a continuous assessment at various points along the epidemiological curve.

### Socioecological Model

**Figure 2.**
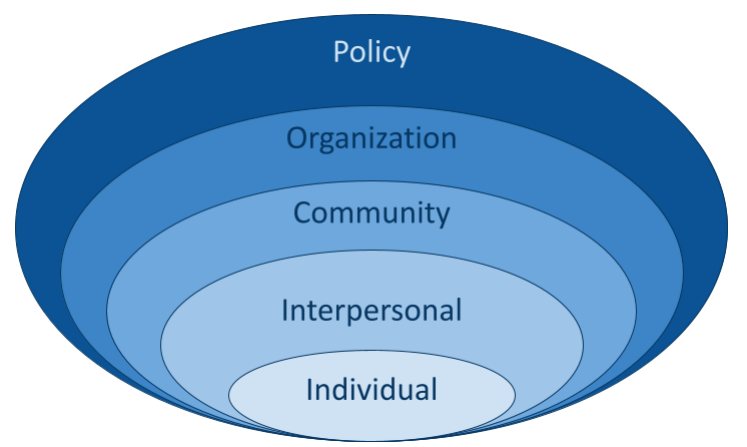
Socioecological model.

The socioecological model illustrates the health impacts of various components of society and the environment (Figure 2) [18]. Given its widespread application in health promotion and public health [19-22], it is important to evaluate its use in health misinformation. Counterinfodemic activities fit within this paradigm as the information environment is an increasingly recognized determinant of health influenced at multiple levels, from clinical interactions to social media regulation [23]. This perspective indicates a need to comprehensively target misinformation throughout the socioecological spectrum [8], reflected in the US Surgeon General’s “whole of society” response to misinformation [24,25] and reports from the World Health Organization and other public health experts [26,27].

### Environmental Health Framework

Environmental health is an area of public health focused on the health impacts of the natural and built environment. Despite its decades of use, the term “information environment,” previously defined as the space where people receive and process information to make sense of the world [28,29], has only recently been applied to misinformation. In national defense, it was conceptualized to facilitate (often clandestine) information operations [30]. Political science literature has examined to what extent the information environment is conducive to political knowledge, civil discourse, and other democracy-relevant outcomes [31]. In both instances, the implied orientation of the information environment is toward information producers, rather than information consumers.

Environmental analogies about health-related information challenges have expanded, as scholars have alluded to the toxic effects of a polluted media environment [32,33]. In 2021, the US Surgeon General included the subtitle “Building a Healthy Information Environment” in his special advisory on misinformation [24]. The New York City Commissioner of Public Health, Ashwin Vasan, and the New York City Mayor, Eric Adams, recently urged public health authorities to “treat social media as a toxin, ever present in our daily environments” [34]. Here, the implied orientation is toward information consumers.

**Figure 3.**
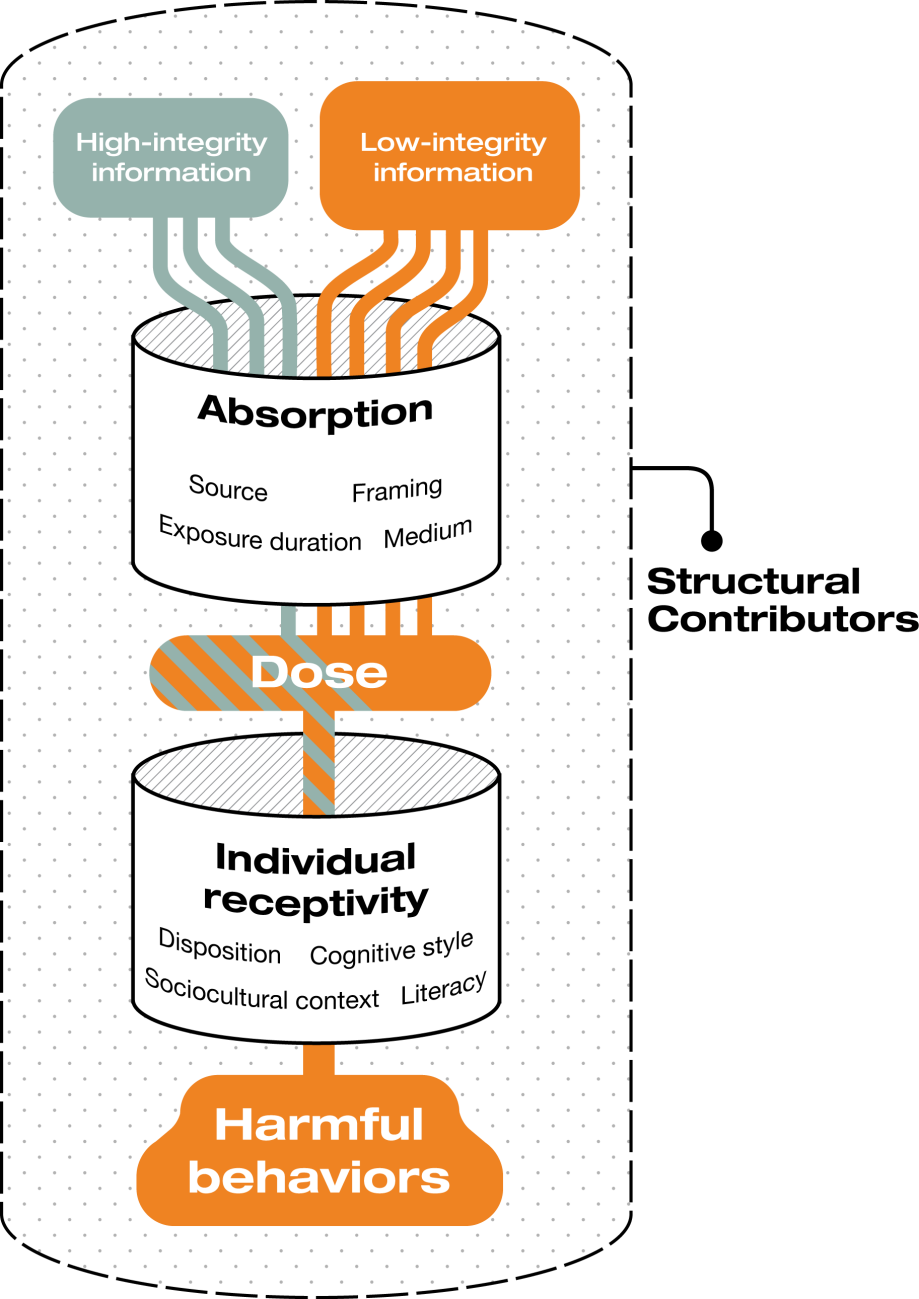
Environmental health framework.

Despite the use of environmental metaphors, environmental health frameworks have been underused to understand public health–related information challenges. From a public health perspective, the information environment has been defined as an adaptive space that includes content from traditional and web-based media and in-person sources and technology to access and process this content [35]. This paradigm highlights several mechanisms of misinformation spread and corresponding opportunities for intervention: altering the dose of information of variable integrity to which an individual is exposed, influencing an individual’s receptivity to toxic misinformation, assessing the threat posed by a claim or narrative (referred to as hazard identification), and mitigating the harms of information hazards through multipronged approaches (hazard management) (Figure 3). Detailed definitions and examples of each of these intervention types are provided in the “Methods” section.

By applying these 3 models, this study sought to identify gaps and opportunities in an aggregate view of pandemic-related infodemic management interventions and to assess the use of public health frameworks to broadly structure and strategize responses to infodemics.

## Methods

### Data Collection

This analysis drew on a dataset of infodemic management interventions aiming to address the effects and spread of misinformation that was previously developed as part of a report commissioned by the National Academies of Sciences, Engineering, and Medicine, which ultimately led to a peer-reviewed publication [8,36,37]. These interventions, which were identified between October 2022 and January 2023, include guidelines, policies, and tools from local and federal governments, public health departments, nonprofits, universities, technology and media companies, and other organizations [8]. The original authors identified these interventions through searches of the following sources: academic literature about infodemics and infodemic management; gray literature from organizations including federal agencies, nongovernmental organizations, and technology companies; and websites from state and local health departments [8,36]. Interviews with key informants were used by the original authors to identify additional interventions [36]. We expanded this dataset to include additional interventions lacking from the original dataset, focusing on interventions performed by professional societies that were identified through a similar search strategy, and reviewing the websites and resources of medical and scientific societies. As many societies’ interventions were undertaken without either a publication or a description of such interventions on the societies’ websites, the goal with this expansion was to be illustrative of these interventions and not exhaustive. The final dataset consisted of 379 interventions and can be made available upon request.

### Data Analysis

We used framework analysis, a form of qualitative content analysis useful for applied health policy research [12,13]. Framework analysis provides a comprehensive and systematic approach to describe, interpret, and identify patterns in policies and procedures [12,14]. Codes based on thematic frameworks are applied to cases, allowing data to be compared across and within cases [13]. Through applying frameworks to a given topic, framework analysis can assess the relevance of public health analogies that are frequently applied to health infodemics but have yet to be rigorously defined in this context. Studying multiple frameworks allows for a more comprehensive lens to examine the many dimensions to an issue such as misinformation [13].

Five steps are involved in framework analysis: (1) familiarization, in which the researchers become immersed in the data and reflect on patterns; (2) identifying the thematic framework, based on emerging themes; (3) indexing, or coding components of the data that correspond to themes; (4) charting, which involves rearranging data based on themes; and (5) mapping and interpretation, when themes are analyzed through the charts [12]. We first familiarized ourselves with the data by reviewing the intervention descriptions and websites in the dataset. The thematic frameworks were identified based on prior literature cited in the introduction that provide a range of perspectives to conceptualize misinformation. We developed a coding scheme of deductive codes drawn a priori from the components of the frameworks. This coding scheme accommodated additional inductive codes that emerged through the coding process.

The epidemiological model included the following codes: prevention, social listening, risk assessment, and response. Prevention activities proactively protect populations and information networks from the adverse effects of an infodemic. Social listening activities identify and track harmful (web-based) narratives [38]. Risk assessments determine which narratives require intervention based on factors such as its spread over time, the channels in which it is disseminated, and the communities it affects, with the goal of avoiding expending limited resources on or giving oxygen to low-impact narratives [38]. For example, narratives about vaccines causing infertility that are disseminated widely in the press and on social media during a pandemic would be considered high risk [38]. Finally, rapid responses curtail the spread of harmful information.

The codes derived from the socioecological model included individual, interpersonal, community, organization, and public policy, referring to the societal level at which influence was exerted on the information environment [19]. A public policy intervention was considered to be “a choice made by government to undertake some course of action” involving goals and means of reaching them [39].

The following codes were applied for the environmental health model: dose (which could be further specified as increasing high-integrity information exposure, decreasing low-integrity information exposure, or influencing absorption), receptivity, hazard identification, and hazard management. Drawing from toxicology, “dose” refers to the concentration of low-integrity information compared with high-integrity information, defined as information that is “trustworthy; distinguishes fact from fiction, opinion, and inference; acknowledges uncertainties; and is transparent about its level of vetting,” [40] and the degree of absorption of this content [41]. Hazard identification and management are conducted by organizational and governmental entities engaged in infodemic management and information integrity protection. Analogously to toxicology approaches, hazard identification refers to assessing the health effects of an information toxin [42]; hazard management describes multipronged approaches to evaluating and mitigating the threats posed by such a toxin. While structural determinants (eg, health care access or socioeconomic marginalization) influence the information environment, we did not code for this domain in order to focus on the individual components of the information environment that are specific to this model. Examples of each of these codes are given in Table 1.

**Table 1.** Example interventions corresponding to each environmental health code.

Code	Examples	Examples
Dose	InVID assists journalists in assessing the reliability of videos on social media, thus facilitating the sharing of high-integrity videos while inhibiting the further spread of low-integrity videos.	[43]
Receptivity	Interland is a game developed by Google that teaches young children to distinguish truths from misinformation on the web.	[44]
Hazard identification	Logically tracks misinformation campaigns to understand threats to national security, corporations, nonprofits, and elections.	[45]
Hazard management	CrossCheck, a program run by First Draft, promotes collaboration and resource sharing for journalists responding to misinformation. The Vaccination Community Navigator Program similarly takes a multipronged approach in educating community health workers to boost vaccine confidence.	[46]

Coding was conducted in an iterative, discursive process. One author (JNJ) coded the entire dataset in batches, documenting evolving code definitions and interpretations of the data, where relevant, multiple codes were applied to the same intervention. After each batch, 2 of the authors met to discuss uncertainties and insights that arose, such as ambiguities in the code definitions and emerging patterns in the data. Coding was conducted iteratively, until thematic saturation was reached [47]. Then, DS independently coded a random sample of approximately 20% of the dataset. Codes were reviewed to ensure alignment and discrepancies were resolved through discussions between both authors.

## Results

### Overview

Overall, 379 interventions were included in the final analysis, including 14 interventions from professional societies that were identified through the expanded search. The 3 frameworks lended distinct insights into the functions and capacities of the interventions (Table 2). The applications of each of the frameworks are described in detail in the subsequent sections. For further details on the coding results and representative interventions, see Multimedia Appendix 1.

**Table 2.** Insights from the 3 frameworks.

Key finding	Framework	Supporting evidence	Infodemic management recommendations
Risk assessments are often value-based or poorly defined.	Epidemiological framework	Vague or absent language about how risk assessments are conducted.	Risk assessments should be rigorous, objective, and transparent about how community values are incorporated into decision-making.
Interventions are skewed toward acting at the individual level and often focus on only 1 level of influence.	Socioecological model	Most interventions were focused on either individuals alone or individual members of organizations, rather than implementing structural change with community, interpersonal, organizational, or policy interventions. Only 11% of interventions received more than 1 socioecological code.	Interventions acting at the interpersonal, community, organizational, and policy levels should be explored, and structural barriers to implementing interventions at these levels should be identified and overcome. Collaborations should involve interventions targeting multiple levels of the socioecological spectrum.
Interventions often lack mechanisms to reach their intended audiences (ie, the Field of Dreams Fallacy) [48].	Socioecological model	Abundance of resources and tools that lacked connections to established workflows and organizations within the socioecological spectrum.	Interventions should be developed in partnerships with the organizations that are intended to use them.
Interventions place a greater emphasis on increasing high-integrity information rather than decreasing low-integrity information.	Environmental health framework	More than 3 times as many interventions address high-integrity as low-integrity content.	Interventions that decrease the spread of low-integrity information should be developed.
Demographic factors are emphasized when addressing receptivity to misinformation, while psychological factors are overlooked.	Environmental health framework	Focus on targeting racial, cultural, or age-related communities.	Interventions should consider approaches to segmenting audiences based on personas and psychobehavioral factors.
Interventions that address receptivity tend to involve a one-time action rather than longitudinal education.	Environmental health framework	Prevalence of self-contained courses, games, handouts, etc, that lack mechanisms to reinforce instruction over time.	Media literacy initiatives should incorporate mechanisms for longitudinal instruction on detecting and responding to misinformation.
Few organizations are equipped to implement hazard management approaches, despite increasing awareness that such approaches are critical.	Environmental health framework	Overrepresentation of tool kits, handbooks, and other resources lacking direct action in the hazard management category.	Media, public health, and government agencies should adopt hazard management approaches.

### Epidemiological Framework

By distinguishing between the stages of an infodemic, the epidemiological framework highlighted critical distinctions in the foci of interventions that emerged in response to a specific ongoing or predicted infodemic. This framework was less relevant to interventions that addressed general components of misinformation that were agnostic of a particular crisis, such as tools providing assessments of the credibility of information sources. The framework also did not apply to interventions that lacked a clear audience or mechanism of impact.

In total, 50% (189/379) of interventions were engaged in activities intended to prevent an infodemic itself, in contrast to preventing an individual from falling for misinformation amid an ongoing infodemic. Prevention activities were most prominent when the amount of misinformation was low. Moreover, 19% (73/379) of interventions conducted social listening, monitoring conversations, concerns, claims, and news, online or offline [49]. Social listening tools most often analyzed social media feeds and datasets. The degree of analysis varied widely, from tracking misinformation with artificial intelligence to descriptive statistics on rumor spread. Seven percent (28/379) were risk assessment interventions that assessed the severity or status of an infodemic to inform whether and to what extent a response was needed. These interventions not only provided data that could be relevant to a risk assessment, such as the amount of spread of a rumor, but conducted the risk assessment itself. Most interventions (286/379, 76%) responded to an ongoing infodemic, primarily through fact-checking, debunking, and amplifying reliable information and sources. They also conducted prebunking to address topics for which misinformation is already widespread.

### Socioecological Model

The socioecological model allowed for a better understanding of the key groups and audiences that are affected by or are in a position to address misinformation. Applying this framework revealed a skew toward interventions that acted at the individual level, rather than the interpersonal or community levels. While most interventions were directed toward organizations, they required exposure or uptake by individual members, rather than spurring structural change within the organization overall. In addition, interventions often lacked a clearly defined target group and means of reaching this audience. By revealing these shortcomings, the socioecological model shed light on opportunities to align valuable resources with the groups with the greatest capacity to leverage them.

We identified 150 (40%) interventions that acted at the individual level. These interventions included media literacy and prebunking initiatives, repositories of reliable information, fact-checks and debunks, and tools evaluating the credibility of claims and sources, when these tools were intended for use by the general public. Interventions acting at the interpersonal level, such as an app that provides guidance about discussing vaccines with friends, were the least common, representing only 2% (9/379) of this dataset. Eleven percent (42/379) of interventions were community-level, targeting groups based on educational systems, geographic regions, and racial or ethnic identities, as well as social networks. The interventions often included content or dissemination strategies tailored to a community’s needs. The 178 (47%) organization-level interventions primarily provided resources and tools that were intended for members of a profession, such as journalists, researchers, physicians, teachers, librarians, policy makers, or organizational bodies. These resources included infodemic management tool kits, communication materials, social listening platforms, media literacy curricula, reporting guidelines, and social media policies. There were 39 (10%) public policy interventions. Most of these policies were developed by federal governments. Two came from the United States; other regions included Singapore, Australia, the United Kingdom, France, Egypt, Germany, and the European Union.

### Environmental Health Framework

The environmental health framework allowed for a more nuanced perspective on the mechanisms through which interventions interacted with the information environment. By outlining a variety of components that contribute to the information environment, this framework underscored the importance of contextualizing misinformation within information networks and audiences.

Most interventions (244/379, 64%) targeted the dose of high- and low-integrity information. More interventions increased the amount of high-integrity information (155/379, 41%) rather than decreasing the volume of low-integrity information (44/379, 12%). We identified 61 (16%) interventions that addressed receptivity to misinformation. Most of these interventions involved media literacy education, including curricula, games, infographics, and web-based courses. Seventeen percent (65/379) of interventions conducted hazard identification by assessing the dose or toxicity of misinformation. These interventions were primarily resources and tools for professionals, particularly infodemic managers, public health communicators, and journalists. The interventions involved content verification, social listening, credibility assessments, and fact-checking. Seventy (19%) hazard management interventions took a comprehensive and higher-level approach to addressing misinformation that went beyond any 1 particular intervention. They often took the form of tool kits, handbooks, field guides, and frameworks intended to inform professional hazard management activities, rather than conducting hazard management themselves.

### Crosscutting Insights

We identified several findings that suggest opportunities for future interventions relating to the use of technology, coordination, and sustainability that surfaced from a combination of all 3 frameworks (Table 3). For example, some interventions such as artificial intelligence–powered chatbots suggested an overzealous application of new technologies that lacked grounding in user needs. Perhaps owing to the urgent and unprecedented nature of the COVID-19 pandemic, interventions were often duplicative and short-lived.

**Table 3.** Crosscutting insights.

Key finding	Supporting evidence	Infodemic management recommendations
Greater strategic direction to align theories of change with desired impact is needed.	Unclear distinctions between efforts to address acute compared with endemic misinformation as well as efforts engaged in prevention versus response. The intended audiences of interventions also tended to be poorly defined.	Interventions should specify the nature of the infodemics they are intended to address, intentionally select a guiding framework, and address the unmet needs of a specific audience.
Technological tools are often built and used without adequate need finding.	Prominence of tools such as chatbots enabled by technology that do not clearly fill a well-defined need.	The design process for interventions should center around identified needs rather than the tool.
Lack of coordination or pervasive duplication of efforts.	Very few initiatives included cross-sector collaboration; those that did were not sustainably funded to persist beyond the pandemic. A number of initiatives duplicate work and effort (eg, see “tool kits”).	Sustainable cross-disciplinary or sector coordination mechanisms may be required to support effective and ethical infodemic management initiatives [50].
Short-term funding opportunities early on in the COVID-19 pandemic were not conducive to long-term sustainability.	Many interventions had concluded or had websites that had not been recently updated.	Sustainability given funding trends should be a key consideration when developing interventions. Funding programs should include support to sustain efforts beyond immediate crises and collect longitudinal data.
The role of incidental information exposure compared with intentional information consumption was rarely accounted for.	Interventions frequently made unsupported assumptions about the degree of agency individuals have in the information they encounter.	Future frameworks should incorporate the distinction between incidental information exposure and intentional consumption.

## Discussion

In our analysis, the epidemiological, socioecological, and environmental health frameworks shed light on trends, gaps, and opportunities among counterinfodemic interventions. The epidemiological framework revealed an opportunity to implement more robust and transparent risk assessment measures in partnership with communities to triage rumors and allocate resources, particularly as more evidence emerges on the threats posed by various claims and narratives. By relying on value judgments, the risk assessments in the interventions in this dataset risk undermining trust and expending limited resources on low-impact efforts. Instead, the World Health Organization recommends developing risk assessment matrices that synthesize considerations such as the timing of a narrative, its spread on various platforms, and the impacted communities to categorize narratives as high, moderate, or low risk, and positive sentiment [38].

The socioecological framework demonstrated the need to target higher levels of influence through collaborations spanning multiple levels, reinforcing a finding from the original analysis of this dataset [8]. Scholars have recently argued that the outsized attention given to individually framed behavioral interventions “pollutes” the discourse and diverts attention from structural interventions [51,52]. This trend was replicated in our dataset, where structural change through public policy or enduring platform adjustments was rarely the priority. As with other complex public health challenges such as diabetes or drug overdoses, structural-level interventions coordinated with efforts acting at other levels of the socioecological spectrum are likely to be more effective and sustainable than individual-level efforts in the case of infodemic management. Policy efforts to protect children from social media–related harms have garnered significant attention, most notably in the US Surgeon General’s recommendation to display warning labels on social media [53]. Despite their limitations, related legislation, such as the Stop Addictive Feeds Exploitation [54], offers potential models for analogous efforts to mitigate the harms of digital infodemics

The socioecological framework additionally revealed the importance of avoiding the Field of Dreams Fallacy [48], as many interventions neglected to specify mechanisms to reach their intended audiences. While the speed of a response is often prioritized in an emergency, the resulting lack of alignment with existing efforts may prove harmful in infodemic management due to the resource and trust barriers to maintaining strong relationships with community partners. Sustaining proactively developed partnerships is needed to increase the uptake and sustainability of infodemic interventions, particularly the tool kits and other resources that were often deployed independently of established partnerships in this dataset.

**Figure 4.**
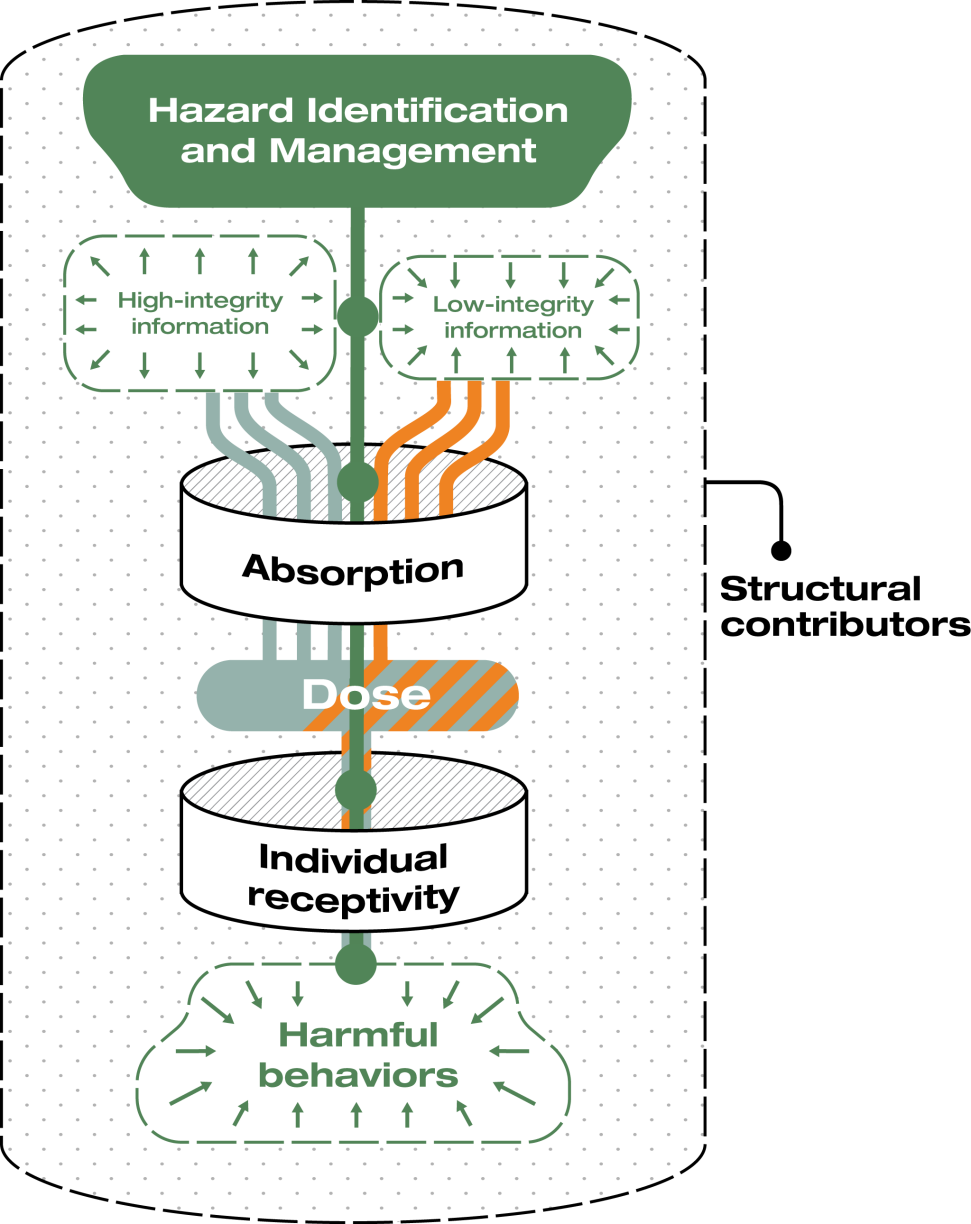
Opportunities for intervention based on the environmental health framework. Points of intervention within this framework are represented by green nodes; for example, interventions can modify individual receptivity to misinformation. The shift in the composition of the information environment toward high-integrity information and subsequent reduction in harmful behaviors as a result of these interventions is indicated with green arrows. Structural contributors influence these dynamics but were not a focus of the present analysis.

The environmental health framework provided a structure for systems-level, multipronged approaches that influence the information environment as a whole (Figure 4). A key finding was that reducing exposure to low-integrity information, which digital platforms can implement through content moderation, deplatforming, and algorithmic adjustments, was a notable gap. Amid the growing politicization of content moderation, many social media platforms have recently rolled back these efforts [11,55]. Differing perceptions of trustworthiness and integrity may also reduce the efficacy of content moderation or even lead to further polarization [6,56]. Regulating algorithmic recommendation and amplification may encourage platforms to prioritize high-integrity content while protecting First Amendment rights [57]. While the answer to bad speech was once considered to be “more speech” [58], in the social media era, it is now recognized that freedom of speech does not equate to freedom of reach [59]. Current revenue models incentivize platform architectures and algorithms that promote content that provokes negative emotional reactions, particularly anger [60].

While many interventions used demographic characteristics to target the information environments of particular communities, psychobehavioral segmenting may allow for more precise tailoring of messages to individuals uniquely receptive to misinformation (eg, those who engage in absolutist thinking) [61,62]. An information environment perspective additionally suggests that initiatives based on inoculation theory could expand their impact through longitudinal rather than one-time modes of engagement and by reaching a saturation point that displaces low-integrity information. Hazard management approaches are critical to address an issue as pervasive as an infodemic. Such approaches were uncommon in our dataset, however, likely due to the funding, coordination, and sustainability challenges. Strong governance and financial support are needed to enable key stakeholders, including media, public health, environmental scientists, and government, to create and sustain hazard management approaches, potentially following models such as the Elections Infrastructure Information Sharing and Analysis Center [50].

Several key crosscutting considerations emerged (Table 3). Infodemic management interventions could benefit from greater strategic direction regarding the theories of change applied in various settings. The intended mechanism and audience of an intervention should be informed by a framework that aligns with the relevant type of information distortion. For example, while misinformation is often considered as part of acute infodemics, endemic misinformation unrelated to particular health events may require different theories of change, use of alternate frameworks (eg, socioecologic or environmental), and corresponding interventions. Too often, the development of tools using novel technologies such as generative artificial intelligence centered the technology itself, rather than a need they are intended to address. Need-finding processes must be incorporated into the design of technologically enabled interventions to maximize their potential impact. Design-thinking principles, for example, provide an approach to explore stakeholders’ needs and develop tailored solutions [63].

Funders and stakeholders involved in the interventions were often fragmented and uncoordinated, leading to duplication and unstrategic allocation of resources. Well-governed and funded coordination mechanisms, perhaps modeled on Elections Infrastructure Information Sharing and Analysis Center, offer an opportunity to streamline resources while diversifying efforts. Since many efforts to counter the COVID-19 infodemic were not sustained after the immediate threat of the pandemic subsided, funding structures that support longitudinal and crisis-agnostic efforts are needed. Interventions rarely accounted for the distinction between incidental exposure and intentional information consumption. While a consumptive lens suggests that individuals make conscious decisions about the information they encounter, from an exposure-based perspective, individuals are subject to influence by information within their environments. Incorporating this distinction into future frameworks may illuminate new approaches for interventions. Overall, by testing these frameworks in our dataset, we identified their strengths and weaknesses, allowing for iterative adaptation to the infodemic management context.

Our analysis was limited in that not all components of the interventions that we considered, such as reach and distribution, were typically reported. As a result, it was sometimes necessary to make inferences about goals and impacts. Many interventions lacked information about time and scale, which resulted in organizing the data in a way that gave the same prominence to small- and large-scale initiatives. This lack of information biased the data toward smaller-scale initiatives, although large-scale initiatives likely had a broader impact. Many of the codes we applied were subjective, not mutually exclusive, and reliant on interpretation, a limitation that was exacerbated when details of an intervention were not available. For example, for the epidemiological framework, prevention and response entail critically distinct activities, but we were unable to distinguish between these 2 foci when information about the stage of the infodemic at which an intervention was deployed was not provided. There was also at times overlap in the insights derived from each framework; our analysis attempted to focus on the dominant framework that surfaced a given insight. The dataset used in this study is not exhaustive; notably, given the focus on terms such as “infodemic management,” a term that emerged during the COVID-19 pandemic, interventions that predate the pandemic may have been underrepresented. Our study was designed to be illustrative, not exhaustive, so it did not use systematic search criteria. This study considered only 3 frameworks, which were chosen based on their prominence in public health and misinformation discourse; future work should consider additional frameworks to illuminate further findings. For example, recent work has adapted a public health prevention framework to infodemic management [64]. Finally, we acknowledge that the feasibility of our recommendations may be limited given resource constraints and an evolving evidence base.

In this study, we used a framework analysis using 3 public health frameworks to illuminate emphases and gaps in interventions to address the COVID-19 infodemic. While many opportunities to expand the reach and impact of interventions were identified, it was also clear that the landscape of infodemic management approaches lacks an overarching strategy and entity responsible for coordinating and evaluating activities. In preparation for future infodemics, emphasis should be placed on multisector collaboration, alignment with measurable and meaningful goals, and top-down approaches to determining and implementing strategies.

## Supplementary material

10.2196/67119 Multimedia Appendix 1 Example interventions.

## References

[1] World Health Organization (2020). Managing the COVID-19 infodemic: promoting healthy behaviours and mitigating the harm from misinformation and disinformation. https://www.who.int/news/item/23-09-2020-managing-the-covid-19-infodemic-promoting-healthy-behaviours-and-mitigating-the-harm-from-misinformation-and-disinformation.

[2] Amin K, Ortaliza J, Cox C, Michaud J, Kates J (2022). COVID-19 mortality preventable by vaccines. Peterson-KFF Health System Tracker.

[3] Bruns R, Hosangadi D, Trotochaud M, Sell K (2021). COVID-19 vaccine misinformation and disinformation costs an estimated $50 to $300 million each day.

[4] Knudsen J, Perlman-Gabel M, Uccelli IG, Jeavons J, Chokshi DA (2023). Combating misinformation as a core function of public health. NEJM Catalyst.

[5] Burstin H, Curry S, Ranney ML (2023). Identifying credible sources of health information in social media: phase 2-considerations for non-accredited nonprofit organizations, for-profit entities, and individual sources. NAM Perspect.

[6] Lalani HS, Laine C (2023). The credibility conundrum: can social media companies define credibility for users?. Ann Intern Med.

[7] Smith R, Chen K, Winner D, Friedhoff S, Wardle C (2023). A systematic review of COVID-19 misinformation interventions: lessons learned. Health Aff (Millwood).

[8] Sundelson AE, Jamison AM, Huhn N, Pasquino SL, Sell TK (2023). Fighting the infodemic: the 4 i Framework for Advancing Communication and Trust. BMC Public Health.

[9] Ziemer CT, Rothmund T (2024). Psychological underpinnings of misinformation countermeasures. J Media Psychol.

[10] Broniatowski DA, Simons JR, Gu J, Jamison AM, Abroms LC (2023). The efficacy of Facebook’s vaccine misinformation policies and architecture during the COVID-19 pandemic. Sci Adv.

[11] Appel RE, Pan J, Roberts ME (2023). Partisan conflict over content moderation is more than disagreement about facts. Sci Adv.

[12] Thomson SB, Srivastava A (2009). Framework analysis: a qualitative methodology for applied policy research. J Adm Gov.

[13] Gale NK, Heath G, Cameron E, Rashid S, Redwood S (2013). Using the framework method for the analysis of qualitative data in multi-disciplinary health research. BMC Med Res Methodol.

[14] Goldsmith L (2021). Using framework analysis in applied qualitative research. Qual Rep.

[15] Eadon YM, Wood SE (2024). Combating contamination and contagion: embodied and environmental metaphors of misinformation. Convergence.

[16] Scales D, Gorman J, Jamieson KH (2021). The Covid-19 infodemic—applying the epidemiologic model to counter misinformation. N Engl J Med.

[17] SEARO Regional Office for the South East Asia (2023). Strategic framework for action for strengthening surveillance, risk assessment and field epidemiology for health security threats in the WHO South-East Asia region. https://www.who.int/publications/i/item/9789290210030.

[18] Bronfenbrenner U (1977). Toward an experimental ecology of human development. Am Psychol.

[19] Dahlberg LL, Krug EG (2006). Violence a global public health problem. Ciênc Saúde Coletiva.

[20] McLeroy KR, Bibeau D, Steckler A, Glanz K (1988). An ecological perspective on health promotion programs. Health Educ Q.

[21] Townsend N, Foster C (2013). Developing and applying a socio-ecological model to the promotion of healthy eating in the school. Public Health Nutr.

[22] Mehtälä MAK, Sääkslahti AK, Inkinen ME, Poskiparta MEH (2014). A socio-ecological approach to physical activity interventions in childcare: a systematic review. Int J Behav Nutr Phys Act.

[23] Graham G, Goren N, Sounderajah V, DeSalvo K (2024). Information is a determinant of health. Nat Med.

[24] Murthy V (2021). Confronting health misinformation: the U.S. Surgeon General’s advisory on building a healthy information environment. https://www.hhs.gov/sites/default/files/surgeon-general-misinformation-advisory.pdf.

[25] Murthy V (2021). A community toolkit for addressing health misinformation. https://www.hhs.gov/sites/default/files/health-misinformation-toolkit-english.pdf.

[26] World Health Organization Regional Office for Europe (2022). Digital solutions to health risks raised by the COVID-19 infodemic: policy brief. https://iris.who.int/handle/10665/356315.

[27] Donovan J, Friedberg B, Lim G, Leaver N, Nilsen J, Dreyfuss E (2021). Mitigating medical misinformation: a whole-of-society approach to countering spam, scams, and hoaxes. https://mediamanipulation.org/research/mitigating-medical-misinformation-whole-society-approach-countering-spam-scams-and-hoaxes.

[28] Wanless A (2023). Seeing the disinformation forest through the trees: how to begin cleaning up the polluted information environment. https://www.oecd-forum.org/posts/seeing-the-disinformation-forest-through-the-trees-how-to-begin-cleaning-up-the-polluted-information-environment?utm_source=user_mailer&utm_medium=email&utm_campaign=send_publish_notification_to_follower.

[29] Wanless A (2023). The more things change. https://kclpure.kcl.ac.uk/portal/en/studentTheses/the-more-things-change.

[30] Joint Chiefs of Staff (2012). Joint publication 3-13 information operations. https://defenseinnovationmarketplace.dtic.mil/wp-content/uploads/2018/02/12102012_io1.pdf.

[31] Jerit J, Barabas J, Bolsen T (2006). Citizens, knowledge, and the information environment. Am J Pol Sci.

[32] Phillips W (2019). The toxins we carry. https://www.cjr.org/special_report/truth-pollution-disinformation.php/.

[33] Phillips W, Milner RM (2021). You Are Here: A Field Guide for Navigating Polarized Speech, Conspiracy Theories, and Our Polluted Media Landscape.

[34] Vasan A (2023). We must treat social media like the toxin that it is. https://thehill.com/opinion/technology/4058281-we-need-to-treat-social-media-like-the-toxin-that-it-is.

[35] Shapiro AW, Jacob N (2022). A CERN model for studying the information environment. https://carnegieendowment.org/2022/11/17/cern-model-for-studying-information-environment-pub-88408.

[36] Sundelson A, Huhn N, Jamison A, Pasquino SL, Kirk Sell T (2023). Infodemic management approaches leading up to, during, and following the COVID-19 pandemic. https://centerforhealthsecurity.org/sites/default/files/2023-04/230407-nasempaper.pdf.

[37] Google Sheets Combatting mis/disinfo- best/existing practices. https://docs.google.com/spreadsheets/d/1VjEwkR3tjgcPmd3ve6N03pqiHMF8hMEojUkRWx8QJFc/edit?gid=872458924&usp=embed_facebook.

[38] World Health Organization, United Nations Children’s Fund (2023). How to build an infodemic insights report in 6 steps. https://www.who.int/publications-detail-redirect/9789240075658.

[39] Howlett M, Cashore B, Engeli I, Allison CR (2014). Comparative Policy Studies: Conceptual and Methodological Challenges.

[40] National Science & Technology Council (2022). Roadmap for researchers on priorities related to information integrity research and development. https://web.archive.org/web/20250116082552/https://www.whitehouse.gov/wp-content/uploads/2022/12/Roadmap-Information-Integrity-RD-2022.pdf.

[41] Kirk MA, Deaton ML (2007). Bringing order out of chaos: effective strategies for medical response to mass chemical exposure. Emerg Med Clin North Am.

[42] Fowle III JR, Dearfield KL (2000). Risk characterization handbook. U.S. https://www.epa.gov/sites/default/files/2015-10/documents/osp_risk_characterization_handbook_2000.pdf.

[43] The invid technologies. InVID.

[44] INTERLAND. be internet awesome. Google.

[45] AI powered information advantage. Logically.

[46] CrossCheck: together, now. First Draft.

[47] Saunders B, Sim J, Kingstone T (2018). Saturation in qualitative research: exploring its conceptualization and operationalization. Qual Quant.

[48] Karsh BT, Weinger MB, Abbott PA, Wears RL (2010). Health information technology: fallacies and sober realities. J Am Med Inform Assoc.

[49] Sommariva S, Mote J, Ballester Bon H (2021). Social listening in Eastern and Southern Africa, a UNICEF risk communication and community engagement strategy to address the COVID-19 infodemic. Health Secur.

[50] Lalani HS, DiResta R, Baron RJ, Scales D (2023). Addressing viral medical rumors and false or misleading information. Ann Intern Med.

[51] Chater N, Loewenstein G (2022). The i-frame and the s-frame: How focusing on individual-level solutions has led behavioral public policy astray. Behav Brain Sci.

[52] Maani N, van Schalkwyk MC, Petticrew M, Buse K (2022). The pollution of health discourse and the need for effective counter-framing. BMJ.

[53] Office of the Surgeon General (OSG) (2023). Social media and youth mental health: the U.S. Surgeon General’s advisory. http://www.ncbi.nlm.nih.gov/books/NBK594761.

[54] Gounardes A Establishes the Stop Addictive Feeds Exploitation (SAFE) for Kids act prohibiting the provision of addictive feeds to minors. https://www.nysenate.gov/legislation/bills/2023/S7694/amendment/A.

[55] Myers SL, Grant N (2023). Combating disinformation wanes at social media giants. https://www.nytimes.com/2023/02/14/technology/disinformation-moderation-social-media.html.

[56] Otala JM, Kurtic G, Grasso I, Liu Y, Matthews J, Madraki G (2021). Political polarization and platform migration: a study of Parler and Twitter usage by United States of America Congress members.

[57] Rubinstein I, Kenneth T (2022). Taming online public health misinformation. Harv J Legis.

[58] Brandeis L Whitney v. People of state of California.(supreme court 1927). https://www.law.cornell.edu/supremecourt/text/274/357.

[59] DiResta R (2018). Free speech is not the same as free reach. https://www.wired.com/story/free-speech-is-not-the-same-as-free-reach/.

[60] Munn L (2020). Angry by design: toxic communication and technical architectures. Humanit Soc Sci Commun.

[61] Salvi C, Iannello P, Cancer A (2021). Going viral: how fear, socio-cognitive polarization and problem-solving influence fake news detection and proliferation during COVID-19 pandemic. Front Commun.

[62] Surgo Ventures How do we get America vaccinated?. https://surgoventures.org/vaccine-persona-explainer.

[63] Bender-Salazar R (2023). Design thinking as an effective method for problem-setting and needfinding for entrepreneurial teams addressing wicked problems. J Innov Entrep.

[64] Ishizumi A, Kolis J, Abad N (2024). Beyond misinformation: developing a public health prevention framework for managing information ecosystems. Lancet Public Health.

