# Supplementary material for: Understanding Interventions to Address Infodemics Through Epidemiological, Socioecological, and Environmental Health Models: Framework Analysis
Source: JMIR Infodemiology. 2025 Mar 24;5:e67119. doi: 10.2196/67119 (PMC11957468; doi:10.2196/67119)
Supplement: Multimedia Appendix 1 [file infodemiology-v5-e67119-s001.docx]

## Supplemental Table 1: Example interventions.

| **Framework** | **Code** | **Example(s)** | **Frequency** |
| --- | --- | --- | --- |
| Epidemiological | Prevention | The Johns Hopkins Public Health On Call podcast amplifies factual information about health topics that are not subjects of widespread infodemics, such as ringworm and malaria in the U.S. | 50% |
| Epidemiological | Social listening | The WhatsApp channel What’s Crap on WhatsApp? provides fact-checks based on user-submitted content, and this content was intended to be analyzed to understand the characteristics of circulating misinformation in South Africa. | 19% |
| Epidemiological | Risk assessment | The Network Contagion Research Institute developed models to predict vaccine reluctance based on social media trends. | 7% |
| Epidemiological | Response | iHealthFacts is a website that shares fact-checks about health claims on social media. | 76% |
| Socio-ecological | Individual | The browser extension Trusted Times provides assessments of media bias and identifies unreliable news. | 40% |
| Socio-ecological | Interpersonal | Live Chair Health and Shots at the Shop trained barbers or beauty stylists to communicate with their patrons about health topics. The mobile app Voices for Vaccines is a digital guide for individuals to engage in science-based conversations with family and friends about vaccination. | 2% |
| Socio-ecological | Community | The United Nations Children’s Fund (UNICEF) produced a song promoting measures to reduce the spread of Ebola in Liberia in partnership with local musicians using a style of music popular among Liberians. | 11% |
| Socio-ecological | Organization | The First Draft prebunking guide is written for organizational actors such as reporters, fact checkers, governments, but the prebunks created as a result of the guide are delivered to individuals. | 47% |
| Socio-ecological | Public policy | A proposed reform to Section 230 of the Communications Decency Act, among other policies in this dataset, aimed to regulate content on social media platforms. Other policies, in France and Egypt, prohibited media organizations from spreading misinformation. Government initiatives also called for media or digital literacy education. In Singapore, the government used daily text messages to inform the public about the Covid-19 pandemic. | 10% |
